# Simple But Efficacious Enrichment of Integral Membrane Proteins and Their Interactions for In-Depth Membrane Proteomics

**DOI:** 10.1016/j.mcpro.2022.100206

**Published:** 2022-01-25

**Authors:** Pornparn Kongpracha, Pattama Wiriyasermkul, Noriyoshi Isozumi, Satomi Moriyama, Yoshikatsu Kanai, Shushi Nagamori

**Affiliations:** 1 Department of Laboratory Medicine, The Jikei University School of Medicine, Tokyo, Japan; 2 Department of Collaborative Research for Biomolecular Dynamics, Nara Medical University, Nara, Japan; 3 Department of Bio-system Pharmacology, Graduate School of Medicine, Osaka University, Osaka, Japan

**Keywords:** transmembrane domain, transporter, urea, alkaline, protein complex, protein–protein interaction, ACN, acetonitrile, AMED, Japan Agency for Medical Research and Development, BCA, bicinchoninic acid, FA, formic acid, GO, Gene Ontology, GRAVY, grand average of hydropathy, HEK293T, human embryonic kidney 293T cell line, IPA, ingenuity pathway analysis, MS, mass spectrometry, PPI, protein–protein interaction, PSM, peptide spectra match, SDB-XC, styrene-divinylbenzene crosslinked, SDC, sodium deoxycholate, SLC, solute carrier, STRING, Search Tool for the Retrieval of Interacting Genes/Proteins, TMD, transmembrane domain, Tris, Tris(hydroxymethyl)aminomethane

## Abstract

Membrane proteins play essential roles in various cellular processes, such as nutrient transport, bioenergetic processes, cell adhesion, and signal transduction. Proteomics is one of the key approaches to exploring membrane proteins comprehensively. Bottom–up proteomics using LC–MS/MS has been widely used in membrane proteomics. However, the low abundance and hydrophobic features of membrane proteins, especially integral membrane proteins, make it difficult to handle the proteins and are the bottleneck for identification by LC–MS/MS. Herein, to improve the identification and quantification of membrane proteins, we have stepwisely evaluated methods of membrane enrichment for the sample preparation. The enrichment methods of membranes consisted of precipitation by ultracentrifugation and treatment by urea or alkaline solutions. The best enrichment method in the study, washing with urea after isolation of the membranes, resulted in the identification of almost twice as many membrane proteins compared with samples without the enrichment. Notably, the method significantly enhances the identified numbers of multispanning transmembrane proteins, such as solute carrier transporters, ABC transporters, and G-protein–coupled receptors, by almost sixfold. Using this method, we revealed the profiles of amino acid transport systems with the validation by functional assays and found more protein–protein interactions, including membrane protein complexes and clusters. Our protocol uses standard procedures in biochemistry, but the method was efficient for the in-depth analysis of membrane proteome in a wide range of samples.

## Highlights

• Fractionation of membranes improves the identification of membrane proteins.
• Membranes washed with urea or alkaline increase identified transmembrane proteins.
• Urea wash increases the detection of multispanning transmembrane proteins.
• Proteomics of urea-washed membranes keeps more protein–protein interactions.

## Graphical Abstract

**Figure:**
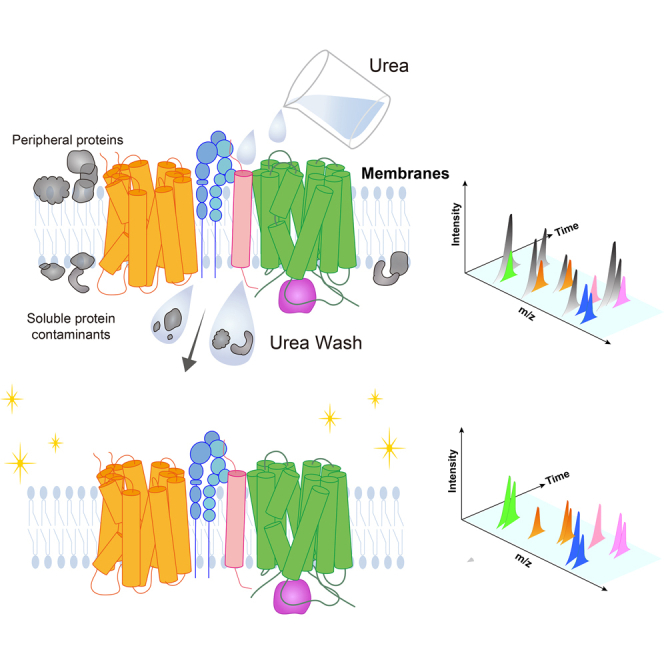

## In Brief

Membrane proteins, particularly integral membrane proteins, are barely detected in bottom–up proteomics because of their complex nature and abundant soluble proteins. We applied standard biochemical procedures to optimize the sample preparation method for membrane proteome. Membranes were precipitated by ultracentrifugation, followed by treatment with urea or alkaline solutions to remove contaminants. This enrichment was critical to obtain comprehensive membrane proteome data. Among the methods, washing membranes by urea distinctly revealed intricate membrane proteome with keeping protein–protein interactions.

Approximately 30% of eukaryotic genes were predicted to encode membrane proteins (1). Membrane proteins play a variety of roles in crucial biological functions, for example, transport of biological substances (ions, nutrients, metabolites, and signaling molecules), signal transduction, bioenergetic processes, immune response, cell adhesion, and cell–cell interaction. Membrane proteins, or more precisely integral membrane proteins, have hydrophobic surfaces that allow the proteins to insert deeply into lipid bilayers. These membrane proteins are challenging to study with biochemical methods because they are presented in low levels and unstable outside the lipid bilayers (2, 3). On the other hand, soluble proteins, such as cytoplasmic proteins or peripheral membrane proteins, are relatively abundant and, in many cases, more stable than membrane proteins in test tubes. Despite the significance of membrane proteins in biological systems, numbers of membrane protein studies have not been greatly denoted because of difficulties in handling and detection.

Mass spectrometry (MS)–based proteomics has become a standard analytical tool in various biological research fields, from basic research to medical applications (4). The presence of proteomics is increasing in the field of membrane protein research, revealing critical physiological roles and identifying disease-related biomarkers and drug targets (5, 6). While bottom–up proteomics is one of the most potent methods to explore protein molecules comprehensively, membrane proteins are often underrepresented in such proteome data because of the difficulties of detection by any means as described previously. It is yet necessary to meet the growing demand for improving the quality and quantity of membrane proteomics data, although thousands of great efforts have been improving membrane proteomics. Several steps are essential to enhance the efficacy of membrane proteome analysis: optimization of the sample preparation, inventions of mass spectrometers, and development of data processing and analysis (7, 8, 9). For instance, great achievements were shown in methods for solubilization of membrane proteins or efficient peptide digestion (3, 9, 10, 11, 12, 13). An elevating column temperature of LC is very critical to recovery peptides from integral membrane proteins (14). Multistep or high-resolution chromatography has contributed significantly to the comprehensiveness of proteomics, resulting in the identification of many membrane proteins (15, 16). Also, combinational approaches have been successful (13, 17).

In this study, we focused on the initial step of the sample preparation. Based on previous biochemical studies of membrane proteins, it is well known that membrane protein enrichment helps to overcome the low abundance of membrane proteins. To explore the function and structure of membrane proteins, researchers have isolated membrane vesicles or purified the protein molecules to improve the signal-to-noise ratio in their assays by reducing unexpected contaminations from the soluble proteins (3, 13, 18, 19). Therefore, as researchers have performed in the field of membrane protein research, we also applied ultracentrifugation to enrich membranes in the sample preparation steps of proteomics. However, because of the low abundance of membrane proteins, cytoplasmic proteins and peripheral membrane proteins interfere with the detections of membrane proteins even in membrane fractions. These contaminants attach to the integral membrane proteins or the lipid bilayers by hydrophobic, electrostatic, or noncovalent interactions. Therefore, a method to clean the contaminants, “membrane washing”, is another critical step in enriching membrane proteins (20). Membrane fractions are often treated with hypotonic solutions or high ionic strength buffers (18, 21, 22). In the proteomic analysis by MS, several studies employed the high pH sodium carbonate called alkaline-wash or treatment (21, 23). Alternatively, in the field of membrane protein biochemistry, researchers have used urea solution to clean membranes for functional assays of membrane proteins (18, 19, 24, 25). Despite the prevalent applications of membrane washing methods, as far as we know, the precise advantages and details have not yet been demonstrated in membrane proteomics. Although our group and another researcher group have applied urea-treatment to membrane proteomics (26, 27, 28), there is no characterization of urea-treatment for membrane proteomics and no comparison with other methods, such as alkaline-treatment. Using a human cell line as a model, we compared each step of the membrane enrichment side by side and settled a simple and effective method for “in-depth” membrane proteomics. The evidence in this study shows that our optimized protocol provides deeper and further comprehension of the membrane proteome and improves data quality for quantification.

## Experimental Procedures

### Materials

Sucrose, Tris(hydroxymethyl)aminomethane (Tris), NaCl, CaCl_2_, Dulbecco's modified Eagle's medium, urea, Na_2_CO_3_, sodium deoxycholate (SDC), iodoacetamide, ethyl acetate, acetonitrile (ACN), distilled water, methanol, TFA, ammonia solution, formic acid (FA), triethylammonium bicarbonate (1 M, pH 8), and other chemicals were purchased from FUJIFILM Wako. EDTA was purchased from Nacalai Tesque. Fetal bovine serum and penicillin–streptomycin were purchased from Life Technologies. Protein digestion standard mixture (MassPREP: yeast alcohol dehydrogenase, rabbit glycogen phosphorylase b, bovine serum albumin, and yeast enolase I) was purchased from Waters. Protease inhibitor cocktails and trypsin were purchased from Roche. Tris(2-carboxyethyl) phosphine hydrochloride was purchased from Thermo Fisher Scientific. Styrene-divinylbenzene crosslinked (SDB-XC) and octadecyl (C18) Empore disks were purchased from 3M. Protein LoBind tubes (1.5 ml) were purchased from Eppendorf. Dissolution buffer consists of 6 M urea, 0.1 M Na_2_CO_3_, and 0.5% w/v SDC.

### Culturing of Human Embryonic Kidney 293T Cells

Human embryonic kidney 293T (HEK293T) cells were maintained in Dulbecco's modified Eagle's medium supplemented with 10% fetal bovine serum and penicillin–streptomycin at 37 °C with 5% CO_2_ and humidity. For every experiment, the cells were cultured for 3 days to obtain 90% confluence. For proteomic analysis, cell pellets were washed twice with ice-cold PBS, harvested by centrifugation, quickly frozen in liquid nitrogen, and stored at −80 °C until use.

### Isolation of Whole Cell Lysate

Typically, pellets from 3.0 × 10⁷ HEK293T cells were lysed by sonication on ice for 5 s on/off 10 times with level 8 by a handheld ultrasonic homogenizer (UR-21P; TOMY) in 2.0 ml of 9.8 M urea with protease inhibitor cocktail (cOmplete; Roche) and phosphatase inhibitor cocktail (PhosSTOP; Roche). The clear lysate was collected by centrifugation at 15,000*g* for 30 min at 15 °C, and named “whole cell lysate”. Very little sediment was visible. The protein concentration of the lysate was measured by bicinchoninic acid (BCA) protein assay.

### Isolation of Crude Membrane

The crude membrane was isolated by a centrifugation method as described with some modifications (19). The pellet of HEK293T cells was homogenized on ice for 20 strokes, 150 rpm, in homogenization buffer (20 mM Tris–HCl [pH 7.4], 150 mM NaCl, 250 mM sucrose, 1 mM EDTA, and protease inhibitor cocktail) using a Potter–Elvehjem homogenizer. The homogenate was centrifuged at 1000*g* for 5 min at 4 °C. The supernatant was collected and then centrifuged at 10,000*g* for 10 min at 4 °C. After centrifugation, the supernatant was ultracentrifuged at 438,000*g* for 30 min at 4 °C. The pellet was resuspended in the resuspension buffer to obtain a membrane fraction named “crude membrane”. Crude membrane was also called “No Wash sample”. Protein concentration was measured by BCA protein assay.

### Washing of Crude Membrane

The membrane samples washed with urea solution and alkaline solution are called “Urea Wash sample” and “Alkaline Wash sample”, respectively. For Urea Wash sample, 150 μg of crude membrane of HEK293T cells were dissolved by pipetting in 8 M urea at pH 7 to 7.5 (final 0.2 μg/μl of protein concentration) and then incubated for 20 min at 4 °C. To sediment the membranes, the samples were twofold diluted by ice-cold water and centrifuged at 438,000*g* for 30 min at 4 °C. After the ultracentrifugation, the supernatant was completely removed. The pellet was dissolved by the bath-type sonicator (Branson) for 5 s on/off 10 times in a dissolution buffer (6 M urea, 0.1 M Na_2_CO_3_, and 0.5% w/v SDC) and transferred to a new tube (protein LoBind tube; Eppendorf). For Alkaline Wash sample, 150 μg of crude membrane of HEK293T cells were resuspended by pipetting in ice-cold 0.1 M Na_2_CO_3_ at pH 11 (final 0.1 μg/μl of protein concentration) and incubated for 20 min at 4 °C. The sample was centrifuged at 438,000*g* for 30 min at 4 °C. After ultracentrifugation, the supernatant was completely removed. The pellet was dissolved in the dissolution buffer as described previously and transferred to a new tube. The protein concentration was determined by micro BCA protein assay.

### Sample Preparation and LC–MS/MS Analysis

Twenty micrograms of protein samples (whole cell lysate, crude membrane [No Wash], crude membrane after Urea Wash, and crude membrane after Alkaline Wash) were incubated with 2 mM Tris(2-carboxyethyl) phosphine hydrochloride for 30 min at 37 °C for reduction, followed by alkylation with 55 mM iodoacetamide for 30 min at room temperature. The mixture was then diluted fourfold with 0.1 M triethylammonium bicarbonate (pH 8) and subjected to trypsin digestion (1:40, trypsin:sample ratio) overnight at 37 °C. Then, the digestion was acidified with TFA to be about pH 2. The samples were mixed with a peptide standard mixture (10 fmol/ml MassPREP; Waters) and subjected to the phase transfer surfactant method for SDC removal (9). In brief, 110 μl of ethyl acetate was added to 110 μl of the digested solution, and the mixture was shaken for 1 min, and then centrifuged at 15,700*g* for 2 min to obtain aqueous and organic phases. SDC in organic phase was removed once. Then ethyl acetate at the top layer of the aqueous phase was eliminated by a cold vacuum evaporator for 7 min. The mixture was centrifuged at 20,000*g* for 3 min, and the supernatant was collected and adjusted at about pH 9 with ammonia. For fractionation, StageTips were prepared using two disks of SDB-XC material, washed, and equilibrated with 100% methanol, followed by 100% ACN/0.1% w/v NH_4_OH and 0.1% w/v NH_4_OH. The peptide mixtures were loaded onto the SDB-XC StageTips. The peptides were fractionated with increasing concentrations of ACN (0%, 10%, 20%, and 80%) in 0.1% w/v NH_4_OH. The first fraction was acidified by FA to about pH 2 and subjected to desalting with C18-StageTips (29). The StageTips with one disk of C18 material were washed and equilibrated with 100% methanol, followed by 90% v/v ACN/0.1% v/v FA and 3% v/v ACN/0.1% v/v FA. The peptide mixture was loaded onto the equilibrated C18 StageTips. Peptides were washed by 3% v/v ACN/0.1% v/v FA twice and eluted by 90% v/v ACN/0.1% v/v FA twice. Each fraction was dried under a cold vacuum evaporator and stored at −20 °C within 1 to 3 months until LC–MS/MS data acquisition. For LC–MS/MS measurement, the peptides were dissolved in 10 μl of the measurement buffer (3% v/v ACN and 0.1% v/v FA). Samples were measured using Q Exactive (Thermo Fisher Scientific) with Advance UHPLC (Michrom Bioresources/Bruker) equipped with a trap column (L-column ODS, 0.3 I.D. × 5 mm; CERI) and C18 packed tip column (100 μm I.D. × 15 cm; Nikkyo Technos). Two microliter of the samples was injected into the LC by PAL autosampler injection (CTC Analytics). To obtain the ideal temperature for analyzing hydrophobic peptides (14), the column temperature was controlled at 60 °C using ESCO column oven (AMR). Mobile phases were composed of buffer A (0.1% v/v FA) and buffer B (100% v/v ACN). A gradient condition was configured a 5 to 40% buffer B in 100 min with a flow rate of 400 nl/min. The spray voltage was 2000 V, and the capillary temperature was 275 °C. The full MS/dd-MS^2^ (Top10) method was used. A single full-scan mass spectrum and 10 MS/MS spectra were acquired from each duty cycle to determine peptide molecular weights and amino acid sequences, respectively. The selected conditions for this method were as follows: (i) the full mass range of *m/z* 300 to 1700; (ii) isolation width of *m/z* 3.0; (iii) normalized collision energy of 27%; and (iv) dynamic exclusion of 30 s.

### Data Processing and Analysis

The enriched membranes were prepared from two independent culture batches of HEK293T cells, and each replicate was subjected to two runs of LC−MS/MS. Raw data were analyzed using Proteome Discoverer 2.2 (Thermo Fisher Scientific) with Mascot 2.6.2 (Matrix Science) against UniProt human database (released in March 2019) containing 148,117 protein sequence entries including internal standards and trypsin sequences. The maximum missed cleavage sites were set to 3, precursor mass tolerance was set to 10 ppm, and fragment mass tolerance was set to 0.01 Da. The carbamidomethylation on Cys was set as a fixed modification. The oxidation on Met and deamidations on Asn and Gln were set as variable modifications. The false discovery rate of peptide identification using percolator in proteome was set at 0.01, which was calculated from the target-decoy search approach. For label-free quantification, a combination of Minora Feature Detector, Feature Mapper, and Precursor Ions Quantifier nodes was used in Proteome Discoverer 2.2 based on the manufacturer's manual. Briefly, Minora Feature Detector and Feature Mapper nodes were used with the default settings. The intensity was used for the quantification in Precursor Ion Quantifier node. Normalization between datasets was performed using total peptide amounts. The peptide-level precursor intensity values are summarized into protein-level abundances. Peak intensities of peptide ions correlate well with protein abundances (30, 31, 32). After analysis by Proteome Discoverer 2.2, the protein list was exported as an Excel file. Prior to calculating the number of identified proteins, the peptide standards were excluded. The list categorized the identified proteins into three groups: “High confidence”, “Peak found”, and “Not found”. According to the manufacturer, these categories of proteins were evaluated based on quantification of precursor ions and availability of peptide spectra matches (PSMs) for the protein. “High confidence” proteins mean the precursor ion was detected, and the best confidence of the PSMs for the proteins was determined (false discovery rate of 1%). “Peak found” refers to proteins that showed only the results from precursor ion quantification searches while the PSM was not determined (unidentified spectra). “Not found” refers to proteins that did not exist in a particular sample but were detected in another sample. Unless described separately, the proteins labeled as “identified proteins” in all experiments were determined from only “High confidence” proteins.

### Characterization of Membrane Proteins

The localization and functional categories of all identified proteins were determined by the Gene Ontology (GO) using DAVID Bioinformatics Resources, version 6.7 (33, 34) and the ingenuity pathway analysis (IPA) (released in autumn 2019; Qiagen).

The proteins were annotated by two databases and one algorithm (GO, IPA, and TMHMM). The GO is a common resource describing the localization of gene products and their functions. The GO provides a consistent information-rich terminology applicable across species and information repositories (35). IPA is a knowledge-based software for the analysis, integration, and interpretation of data obtained from omics experiments. TMHMM is a membrane protein topology prediction method based on a hidden Markov model (36). It predicts transmembrane helices and differentiates between soluble and membrane proteins with a high degree of accuracy. A protein was classified as “membrane protein” when it falls in at least one of these cases: (1) GO term, if the protein is described as “membrane” in cellular components; (2) IPA, if the protein was labeled at “plasma membrane”; and (3) TMHMM, if the protein was predicted to possess at least one transmembrane helix domain. Protein interaction and complexes were analyzed by IPA, web resources including Search Tool for the Retrieval of Interacting Genes/Proteins (STRING), a database for protein interaction (37), MINT (The Molecular INTeraction Database) (38), Harmonizome (39), and CORUM (the comprehensive resource of mammalian protein complexes) (40).

To analyze protein–protein interaction (PPI) network, we created two lists by combining 1) 1,573 membrane proteins found in both Urea Wash and Alkaline Wash samples, and 2) proteins specifically identified from each washing method (780 proteins from Urea Wash and 470 proteins from Alkaline Wash). The lists were queried in the STRING database, version 11.5 (37), and the PPI networks were visualized by Cytoscape, version 3.9.0 (41). For the STRING setting, the “physical subnetwork” was selected, the “minimum required interaction score” was set at 0.7, and the “maximum number of interactors to show” was set at none. The clustering option was set at “MCL clustering” with “the inflation parameter: 6”. In Cytoscape, GO term was used for “functional enrichment”. “Singletons” proteins with no interaction were omitted from the displayed Figures.

### Amino Acid Transport Assay in HEK293T Cells

Transport assay was performed as described previously with some modifications (42). HEK293T cells (0.5 × 10^5^ cells/well) were cultured on 24-well poly-d-lysine-coated plates for 3 days. Transport of 10 μM l-[^14^C]leucine (17 Ci/mol; Moravek) or 10 μM [^14^C]glycine (20 Ci/mol; American Radiolabeled Chemicals) was measured for 1 min at 37 °C in Hanks' Balanced Salt Solution (pH 7.4). For the measurement in Na^+^-free buffer, NaCl was substituted with choline chloride. The inhibitors (1 mM 2-aminobicyclo heptane-2-carboxylic acid [BCH] or 1 mM α-methylaminoisobutyric acid [MeAIB]) were added together with the radiolabeled substrates. The cells were lysed, and an aliquot of the lysate was subjected to BCA assay for protein concentration measurement. Radioactivity accumulated in the cells was monitored, and then the uptake values were calculated. Each condition was performed in triplicates.

### Experimental Design and Statistical Rationale

For the comparison of whole cell lysate and crude membrane, the experiment was performed using one batch of HEK293T cells and one technical procedure for sample preparation. In LC–MS/MS analysis, the peptides were measured twice.

For analysis of membrane enrichment by washing methods, the membranes were enriched from two independent batches of HEK293T. The samples from each batch were washed, followed by the sample preparation for LC–MS/MS analysis. Each replicate was subjected to two runs of LC–MS/MS.

For amino acid transport assays, each condition was performed in triplicates from the same culture batch of HEK293T cells. Data are shown as mean ± SEM, n = 3.

Unless indicated separately, all data in LC–MS/MS analysis represent the average numbers ± SD of protein identification calculated from four runs. Statistical significance was calculated with multiple two-tailed *t* test using a two-stage linear step-up procedure of Benjamini, Krieger, and Yekutieli, with Q = 1%. Each condition was compared without assuming a consistent SD. Graphs and statistical significance were analyzed and plotted by GraphPad Prism 8.3 (GraphPad Software). Venn diagram was plotted by the web applications BioVenn (43) and InteractiVenn (44).

### Animal

All animal experiments were designed according to the highest scientific, humane, and ethical principles, and all procedures were approved by the Animal Care and the Use Committee of Osaka University and Nara Medical University.

## Results

### Experimental Workflow

**Fig. 1:**
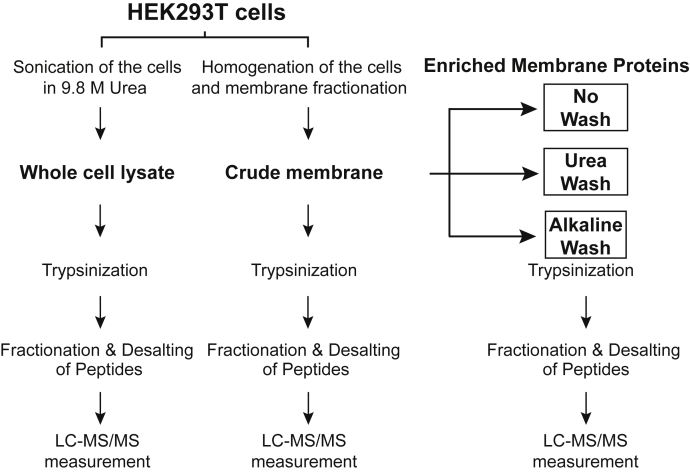
**Membrane enrichment strategy for membrane proteomic analysis of HEK293T cells.** By using HEK293T cells as a model, the sample preparation methods for membrane proteomics were systematically evaluated. The first step is a comparison between whole cell lysate and crude membrane. The crude membrane was then subjected to further membrane enrichment by washing the membranes: (1) no enrichment (No Wash), (2) Urea Wash, and (3) Alkaline Wash. HEK293T, human embryonic kidney 293T cell line.

The goal of this study is to evaluate sample preparation methods for identification and quantifications of membrane proteins by MS. As a benchmark experiment, HEK293T cells were used. In Figure 1, we describe the workflow diagram of experiments in this study. We prepared two types of samples, “whole cell lysate” and “crude membrane”. The crude membrane was divided into three subgroups with further enrichment procedures, called “No Wash” (control), “Urea Wash”, and “Alkaline Wash” as described in the Experimental Procedures section.

### Membrane Isolation Improves Identification of Membrane Proteins

**Fig. 2:**
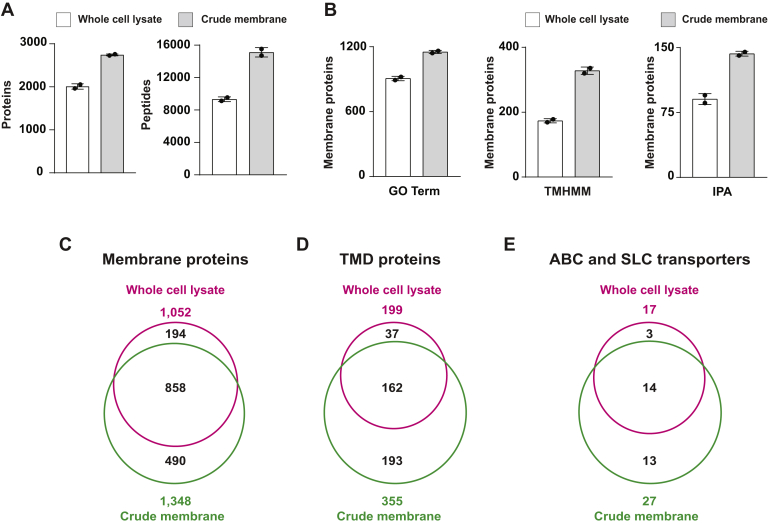
**Comparison of membrane proteome data from whole cell lysate and crude membrane.***A*, bar graphs indicate the identified numbers of proteins and peptides from whole cell lysate (*white*) and crude membrane (*gray*). The data indicate the mean ± SD, n = 2. *B*, bar graphs indicate numbers of membrane proteins annotated by GO, TMHMM, and IPA. The data represent the mean ± SD, n = 2. *C*, Venn diagram indicates the average number of membrane proteins identified in whole cell lysate and crude membrane. *D*, comparison of TMD proteins predicted by TMHMM from whole cell lysate and crude membrane. *E*, comparison of ABC and SLC transporters from whole cell lysate and crude membrane. GO, Gene Ontology; IPA, ingenuity pathway analysis; SLC, solute carrier; TMD, transmembrane domain.

First, we compared the proteome data identified from whole cell lysate and crude membrane. The isolation of membranes by centrifugation resulted in more protein identification (Fig. 2*A*). While we detected 2,008 proteins from the whole cell lysate, 2,743 proteins were identified from crude membrane. The numbers of peptides showed a more drastic difference between the two samples: 9,330 and 15,118 peptides from whole cell lysate and crude membrane, respectively (Fig. 2*A*).

Next, we analyzed the population of membrane proteins from two proteome datasets. Proteins were annotated by using three methods as described precisely in the Experimental Procedures section: GO, TMHMM, and IPA. Briefly, GO is based on a genome database, TMHMM predicts transmembrane domains (TMDs), and IPA is based on knowledge from publications. Annotated by any three methods, the proteome data from crude membrane had more significant numbers of membrane proteins than whole cell lysate (Fig. 2*B*). For convenience with characterizing the proteome data, we defined a protein as “membrane protein” when it was annotated by at least one of the three methods (see the Experimental Procedures section: Characterization of membrane proteins). We detected 1,348 and 1,052 membrane proteins in crude membrane and whole cell lysate, respectively. While 490 proteins are specifically detected in crude membrane, 194 proteins are only from whole cell lysate (Fig. 2*C*). To further evaluate, we focused on the TMD-containing proteins predicted by TMHMM. The TMD proteins were found in 355 and 199 proteins in crude membrane and whole cell lysate, respectively (Fig. 2*D*). The TMD proteins detected only in the crude membrane were over five times higher than the whole cell lysate. In Figure 2*E*, we counted identified numbers of ABCs and solute carriers (SLCs) transporters as examples of membrane proteins possessing multiple TMDs. Generally, transporters are strongly hydrophobic, extremely unstable outside lipid bilayers, and poorly detected by general protocols for MS (2). The ABC and SLC transporters were increased 1.6-fold in crude membrane compared with whole cell lysate. Taken together, the isolation of membranes improved the identification of membrane proteins, especially with proteins containing transmembrane domains.

### Membrane Washing Drastically Increases the Identification of Membrane Proteins

**Fig. 3:**
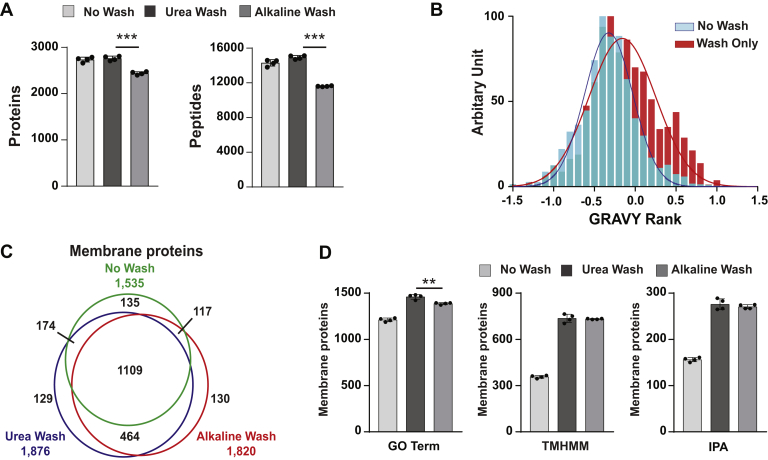
**Evaluation of membrane washing for identification of membrane proteins.** Comparison of identified proteins from No Wash, Urea Wash, and Alkaline Wash samples. *A*, bar graphs indicate the identified numbers of proteins and peptides from each sample. The data indicate the mean ± SD, n = 4, ∗∗∗*p* < 0.001. *B*, evaluation of the protein hydrophobicity by GRAVY distribution. The curves indicate the amounts of proteins with different GRAVY scores ranking ≤0 and ≥0 from the membrane proteins from “No Wash” and “Wash Only” samples (proteins found in either Urea Wash or Alkaline Wash samples, but not in No Wash sample). *C*, membrane proteins annotated by GO, TMHMM, and IPA were merged into one dataset and presented in a Venn diagram to compare three types of samples. *D*, bar graphs indicate the numbers of membrane proteins annotated by GO, IPA, and TMHMM, separately. The data represent the mean ± SD, n = 4, ∗∗*p* < 0.01. GO, Gene Ontology; GRAVY, grand average of hydropathy; IPA, ingenuity pathway analysis.

We found that about 13% of the identified proteins from the crude membrane were predicted as TMD proteins (Fig. 2*B*: TMHMM) and 27 ABC and SLC transporters (Fig. 2*E*). To increase the numbers of detected TMD proteins, we further enriched membrane proteins by means of membrane washing. As shown in Figure 1, we tested three subgroups: nonwashed sample (No Wash sample), sample washed by urea (Urea Wash sample), and sample washed by alkaline (Alkaline Wash sample). The average numbers of identified proteins from four runs of each No Wash, Urea Wash, and Alkaline Wash samples were 2735, 2762, and 2440 proteins, respectively, and of those, 14,307, 14,936, and 11,607 are peptides, respectively (Fig. 3*A*). Alkaline Wash gave significantly fewer identified proteins and peptides than others. About 70% of identified proteins are common among each condition (supplemental Fig. S1, *A*–*C*). All the Pearson's correlation coefficient values were above 0.97, validating that our data are highly reproducible among replicates (supplemental Fig. S1, *D*–*F*).

To characterize the proteome data from the washed membranes, we evaluated the hydrophobicity distribution of membrane proteins in No Wash sample (1,535 proteins) and membrane proteins only found in membrane enriched by Urea Wash or Alkaline Wash (“Wash Only sample”: 723 proteins only found in Urea Wash or Alkaline Wash samples). The hydrophobicity of membrane proteins was calculated by grand average of hydropathy (GRAVY) (45). A higher GRAVY score means higher hydrophobicity. As shown in Figure 3*B*, the distribution of the proteins in Wash Only sample was more hydrophobic than that of No Wash sample, indicating the efficiency of the membrane washing to enrich the hydrophobic proteins, such as membrane proteins. We also compared the GRAVY scores of membrane proteins found in Urea Wash and Alkaline Wash samples. The results showed no significant difference between the two washing methods (supplemental Fig. S2).

Next, we further annotated the proteome data from the three samples. The numbers of membrane proteins in Urea Wash and Alkaline Wash samples were 1.22-fold and 1.19-fold of No Wash sample, respectively (Fig. 3*C*). Approximately one-third of the identified membrane proteins were detected only in Urea Wash sample (593 proteins) or Alkaline Wash sample (594 proteins), whereas 135 proteins were found only in No Wash sample. Almost 64% of the additionally identified proteins (464 proteins) are commonly detected in both Urea Wash and Alkaline Wash samples (Fig. 3*C*). Remarkably, TMD proteins were increased twofold in Urea Wash and Alkaline Wash samples compared with No Wash sample (Fig. 3*D*: TMHMM). We categorized membrane proteins found in Wash Only sample (proteins detected in wash conditions but not in No Wash) based on their molecular functions (Table 1). Washing the membranes enormously improved the identification from wide ranges of protein types, such as enzymes (225 proteins), membrane transport proteins (120 proteins), and receptors (62 proteins).

**Table 1:** Type of proteins found in “Wash Only”

Category	Subtype	Number of proteins
Adaptor/chaperone	—	15
Adhesion molecule	—	20
Enzyme	Kinase	24
	Peptidase	14
	Phosphatase	12
	Other	175
Membrane transport protein	Ion channel	21
	Transporter: ABC^a^	10
	Transporter: ATPase pump^b^	14
	Transporter: SLC^c^	64
	Other^d^	11
Receptor	GPCR	9
	Other	53
Regulator	—	76
Respiration complex	—	32
Translocase	—	10
Vesicle/cargo protein	—	51
Other	—	112

a ABCs.

b ATP-driven pumps.

c SLCs.

d Other: unclassified transporter with high homology to SLC.

**Fig. 4:**
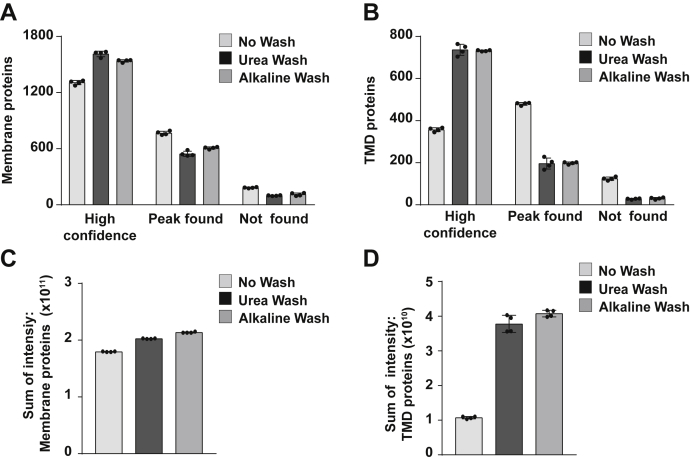
**Impact of washing on the quality of membrane proteome data.** The quality of membrane proteome data is evaluated based on the quantification of precursor ions and the availability of peptide spectra matches (PSMs) for the protein. *A*, the identified proteins were categorized by the quality of quantifying data into three types: “High confidence”, “Peak found”, and “Not found” (see the Experimental Procedures section for the definition). The bar graphs represent the average number of each type in the samples without washing (No Wash) and with Urea Wash or Alkaline Wash. The data indicate the mean ± SD, n = 4. *B*, bar graphs represent the average number of each type in TMD proteins. The data are displayed similar to *A*. *C*, the number of membrane proteins. After normalization by the total peptide amount, the relative abundance of membrane proteins was estimated from the sum of peptide intensity. The bar graphs represent the average sum of peptide intensity in the samples without washing (No Wash) and with Urea Wash or Alkaline Wash. The sum of intensity was calculated from “High confidence” and “Peak found” as defined in the Experimental Procedures section. The data indicate the mean ± SD, n = 4. *D*, the quantity of TMD proteins. The data are displayed similar to *C*. TMD, transmembrane domain.

Membrane proteins, especially TMD proteins, generally have lower abundance, and their mass spectra are hampered by contaminant proteins, resulting in unidentified spectra. Correspondingly, this is a typical problem of the low identification as observed in whole cell lysate and even No Wash membrane. We speculated that the step of membrane washing improves the low quality of the mass spectra from TMD proteins, turning the “Peak found” (unidentified spectra) into “High confidence” resulting in an increase of the protein identification. To test our hypothesis, we calculated the numbers of “High confidence”, “Peak found”, and “Not found” as defined in the Experimental Procedures section, for both membrane proteins and TMD proteins. The results clearly verified our hypothesis. Both Urea Wash and Alkaline Wash intensified the peak quality efficiently, in turn, having extensive “High confidence” proteins and less unidentified spectra, especially significant for the TMD proteins (Fig. 4, *A* and *B*). We summed the intensities of membrane proteins and TMD proteins from “High confidence” and “Peak found” proteins. Sum intensities of membrane proteins and TMD proteins were increased by washing steps (Fig. 4, *C* and *D*). Especially, either Urea Wash or Alkaline Wash enhanced the sum intensities of TMD proteins about fourfold. An increase of peptide precursor ion MS signal intensities correlates with the elevated protein abundance. Collectively, either Urea Wash or Alkaline Wash remarkably amended the membrane proteomics in both quantitative and qualitative manners.

### Urea Wash is the Preferable Washing Method to Detect Multiple TMD Proteins

**Fig. 5:**
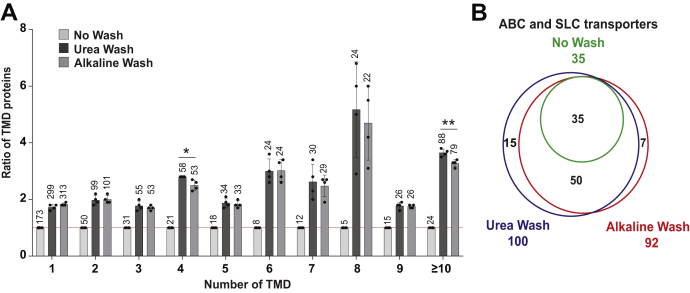
**Effect of washing on the identification of multiple transmembrane domain (TMD) proteins.***A*, comparison of identified TMD proteins from No Wash, Urea Wash, and Alkaline Wash samples. The identified TMD proteins were categorized according to the number of TMDs (x-axis), and the amount of TMD proteins identified from three conditions was counted (numbers above the bars). Then, the values from Urea Wash and Alkaline Wash samples were shown as ratios of No Wash sample. The data are shown as the mean ± SD, n = 4, ∗*p* < 0.05, ∗∗*p* < 0.01. The *red line* indicates the normalized ratio of 1. *B*, Venn diagram represents the ABC and SLC transporters identified in each sample. SLC, solute carrier.

As shown, both Urea Wash and Alkaline Wash significantly improved the identifications of TMD proteins. We further evaluated the proteome data by focusing on TMDs. In total, 961 proteins were predicted to possess at least one TMD (Figs. 3*D* and 5*A*). Both Urea Wash and Alkaline Wash increased the identification of TMD proteins, especially the multiple TMD proteins. Remarkably, Urea Wash significantly increased the numbers of proteins with four or over 9 TMDs in comparison with Alkaline Wash (Fig. 5*A*). Next, we selected ABC and SLC transporters as the representative population of higher-number TMD proteins (46, 47). Compared with No Wash, both Urea Wash and Alkaline Wash made identified these transporters at 2.86-fold and 2.63-fold, respectively (Fig. 5*B*). In terms of the data quality, No Wash exhibited many proteins labeled as “Peak found” whereas both Urea Wash and Alkaline Wash exceedingly increased the numbers of proteins with “High confidence” (supplemental Fig. S3). Both washing also improved the detection of the ABC and SLC transporters (supplemental Fig. S3*C*). Our results indicated the effectiveness of both Urea Wash and Alkaline Wash in the identification of multiple TMD proteins, such as membrane transport proteins. Urea Wash is comparatively better than Alkaline Wash for identification of multiple TMD proteins, regarding the greater numbers of identified multiple TMD proteins (Fig. 5*B* and supplemental Table S1).

Previously, we applied Urea Wash for membrane proteomics of stria vascularis from rat cochleae (26), indicating the usefulness of Urea Wash for proteomics of tissue samples. In this study, we quantitatively compared Urea Wash and Alkaline Wash for the membrane proteomic analysis of brush border membrane vesicles from mouse kidneys (supplemental Fig. S4). We found a similar tendency from the membrane proteome of the tissue sample with the proteome data from the cell line. For example, the numbers of identified proteins and peptides obtained from Alkaline Wash sample were significantly less than those from Urea Wash and No Wash samples (supplemental Fig. S4*A*). All results indicated the merits of the membrane washing step for membrane proteomes of tissue samples and particularly the superiority of Urea Wash over Alkaline Wash.

### Urea Wash Leads to Reveal Profiles of Amino Acids Transport Systems

**Fig. 6:**
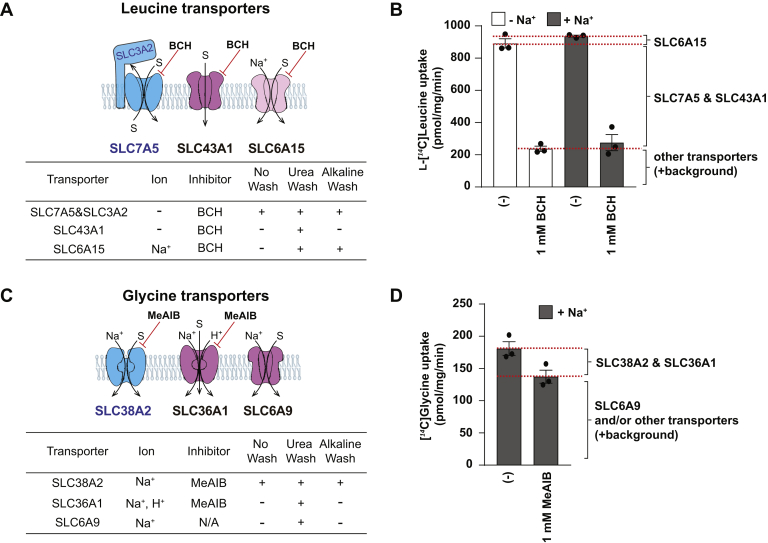
**Urea** W**ash revealed the profiles of amino acid transporters.***A*, leucine transporters and their transport properties (mode of transport, ion requirement, and inhibitors). The table indicates transporter properties and the molecules identified from each enrichment method (No Wash, Urea Wash, or Alkaline Wash). *Blue* represents the amino acid transporters found in all conditions (No Wash, Urea Wash, and Alkaline Wash). *Light pink* represents amino acid transporters found in both washing methods (Urea Wash and Alkaline Wash), and *dark pink* represents amino acid transporters found only in Urea Wash sample. S, substrate. SLC7A5 is an antiporter, SLC43A1 is a facilitative transporter, and SLC6A15 is a Na^+^-coupled symporter. *B*, l-[^14^C]Leucine transport in HEK293T cells. The transport assays were performed in the absence (*white*) or the presence (*dark gray*) of Na^+^ and with or without BCH inhibitor as indicated. The data are shown as the mean ± SEM, n = 3. *C*, glycine transporters and their transport properties. The transporters are displayed similar to *A*. SLC38A2, SLC36A1, and SLC6A9 are Na^+^-coupled symporters. *D*, [^14^C]Glycine transport in HEK293T cells. The transport assays were performed in the presence of Na^+^ and with or without MeAIB inhibitor, as indicated. The data are shown as the mean ± SEM, n = 3. BCH, 2-aminobicyclo heptane-2-carboxylic acid; HEK293T, human embryonic kidney 293T cell line; MeAIB, α-methylaminoisobutyric acid.

Because of the increased identification of transporters by Urea Wash, we applied the proteome data to study transporters. The identified ABC and SLC transporters are listed in supplemental Table S1. Here, we investigated amino acid transporters in a human cell line, HEK293T cells. Leucine is an essential amino acid and a signaling molecule to stimulate cell growth (48). Among known leucine transporters, we detected SLC7A5 in all samples (No Wash, Urea Wash, and Alkaline Wash samples) together with the ancillary subunit SLC3A2 (Fig. 6*A*). By membrane washing, we in addition detected SLC6A15 in both Urea Wash and Alkaline Wash samples and SLC43A1 in only Urea Wash sample. We then performed l-[^14^C]leucine transport assays to evaluate the contribution of each leucine transporter. First, we compared the transport activity in the presence or the absence of Na^+^ because SLC7A5 and SLC43A1 are Na^+^-independent transporters, whereas SLC6A15 is Na^+^-coupled transporter (48, 49, 50). As shown in the bar graph of Figure 6*B*, the uptake activities were similar in the condition with and without Na^+^, indicating SLC7A5 and SLC43A1, but not SLC6A15, are major leucine transporters of the cells. In the presence of 2-aminobicyclo heptane-2-carboxylic acid (BCH), an inhibitor of all identified leucine transporters (48, 49, 50), the uptake was largely diminished confirming the transport specificity of SLC7A5 and SLC43A1 (Fig. 6*B*). Furthermore, Urea Wash samples revealed the profile of the glycine transport system (Fig. 6*C*). SLC38A2 was the only one glycine transporter detected in No Wash sample, whereas SLC6A9 and SLC36A1 were specifically found in Urea Wash sample. Transport of [^14^C]glycine in the presence of Na^+^ showed that 80% of the uptake was resistant to α-methylaminoisobutyric acid (MeAIB), an inhibitor of SLC38A2 and SLC36A1 (51, 52). Most likely, SLC6A9 is the major glycine transporter in HEK293T (Fig. 6*D*).

### Urea Wash Preserves More PPIs

**Fig. 7:**
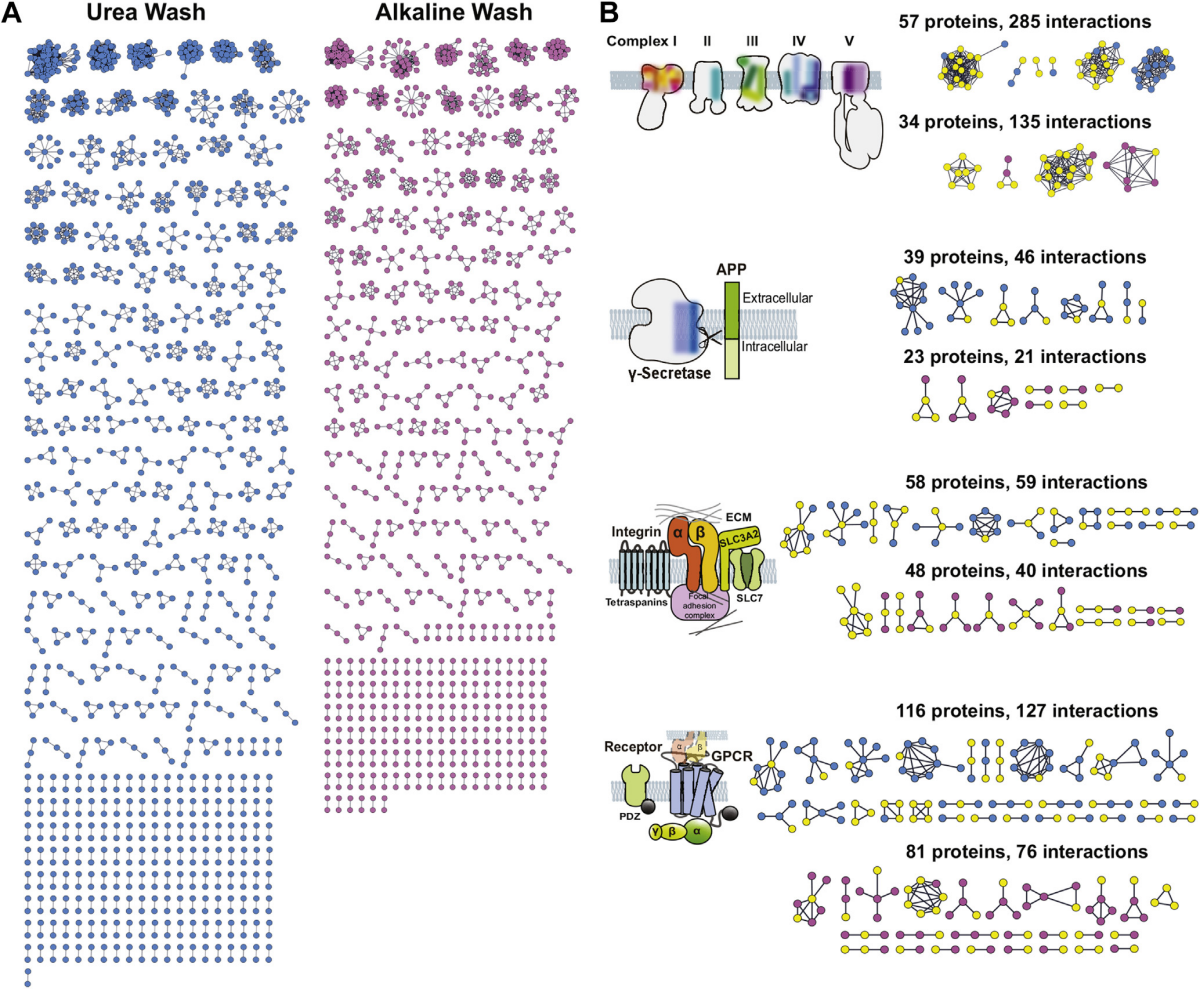
**UreaWash keeps more protein–protein interactions (PPIs).***A*, PPI networks in Urea Wash (*cyan*) and Alkaline Wash (*magenta*) samples. The protein complexes and clusters were analyzed as described in the Experimental Procedures section. Protein molecules are shown as *nodes*, and interactions are indicated as *lines*. *B*, examples of membrane protein complexes/clusters in the PPI networks. *Cyan nodes* represent proteins found in Urea Wash sample (upper row). *Magenta nodes* represent proteins found in Alkaline Wash sample (lower row). The identified proteins that are main components of complexes/clusters are displayed in *yellow* (see full list in supplemental Table S2). *Top panel*, complexes I–V of the mitochondrial respiratory chain. *Second panel*, the γ-secretase complex and its substrate APP (example of type-I transmembrane protein substrates). *Third panel*, membrane protein clusters of integrins and their interacting proteins. Integrins (α and β subunits) mediate the cell adhesion by interacting with their extracellular ligands (extracellular matrix [ECM]) and facilitate the signals *via* the intracellular interaction with the focal adhesion complex. Some integrins interact with the HATs (SLC3A2 and its partner-SLC7 members) and tetraspanin families. *Fourth panel*, clusters of receptor, GPCRs, and their interacting G proteins. A type of GPCR called adhesion GPCRs transinteract with integrins. Some receptors or GPCRs contain a PDZ-binding motif at their cytosolic C termini. GPCR, G proten–coupled receptor; HAT, heterodimeric amino acid transporter.

Many membrane proteins form protein complexes or clusters, and then the complexes/clusters express certain functions (53, 54, 55). We observed that both Urea Wash and Alkaline Wash markedly increased the identification of components in the protein complexes and clusters; for example, in components of respiratory chain complexes were found 19 proteins in No Wash sample, 42 proteins in Urea Wash sample, and 37 proteins in Alkaline Wash sample (supplemental Table S2). We further examined PPI networks containing membrane proteins in Urea Wash and Alkaline Wash samples by using STRING database. To focus on the specific PPI containing membrane proteins in each sample, we merged membrane proteins found in both Urea Wash and Alkaline Wash samples with the identified proteins only in Urea Wash or Alkaline Wash samples and searched the PPI as described in the Experimental Procedures section. As a result, the PPI networks in Urea Wash sample showed more complexes/clusters, nodes (*i.e.* proteins) and interactions than the PPI networks in Alkaline Wash sample (Fig. 7*A*). We found 324 complexes/clusters from 1,271 nodes and 3,591 PPIs in Urea Wash sample (supplemental Table S3), whereas Alkaline Wash sample showed only 276 complexes/clusters from 1,032 nodes and 2,159 PPIs (supplemental Table S4). The ratios of interaction per node were 2.82:1 in Urea Wash sample and 2.09:1 in Alkaline Wash sample.

In addition, we analyzed individual complexes and clusters in detail. One of the top 10 complexes/clusters in Urea Wash sample was respiratory chain complexes (Fig. 7*B*: *top panel*). Many components of each complex were identified in Urea Wash and Alkaline Wash samples (supplemental Table S2). The PPI networks of the respiratory chain complexes showed 57 nodes and 285 interactions in Urea Wash sample and 34 nodes and 135 interactions in Alkaline Wash sample, indicating the preservation of large protein complexes by Urea Wash. Next, we focused on the complexes/clusters at plasma membranes. In the case of a complex with γ-secretase, an enzyme complex that cleaves numerous type-I transmembrane proteins on the plasma membrane (56), we found 39 nodes and 46 interactions in Urea Wash sample and 23 nodes and 21 interactions in Alkaline Wash sample (Fig. 7*B*: *second panel*). The following example is integrins and the proximate proteins, such as heterodimeric amino acid transporters family (SLC3A2 and its partner-SLC7 members) or tetraspanin family, which form protein clusters (57, 58). We found 58 nodes with 59 interactions in Urea Wash sample and 48 nodes with 40 interactions in Alkaline Wash sample (Fig. 7*B*: *third panel*). Receptors, such as some types of G protein–coupled receptors, are also known to form clusters (59). We found 116 nodes with 127 interactions in Urea Wash sample and 81 nodes with 76 interactions in Alkaline Wash sample (Fig. 7*B*: *fourth panel*). Taken all together, although both Urea Wash and Alkaline Wash significantly enhanced identification of membrane protein complexes/clusters, Urea Wash is preferred to detect PPI involving membrane proteins.

## Discussion

We have evaluated membrane enrichment methods for membrane proteomics. The best method tested in this study is composed of two main steps: (1) isolation of membranes by centrifugation and (2) membrane washing with urea. The first step removes nonmembrane fractions roughly, and the later step cleans up contaminants that are attached to membranes. By using the method, we drastically improved the quantity of identified membrane proteins, especially multiple TMD proteins and protein complexes with PPIs, and the quality of the mass spectra.

The isolation of membranes is the first key step for the membrane enrichment. In this study, we selected Potter–Elvehjem homogenization method for the cell disruption because the process is simple and more gentle to the membranes than some harsh methods such as sonication. However, other cell disruption methods, such as high pressure or sonication procedures, would apply if isolation of a specific membrane fraction is required (60). After the cell disruption, we isolated the membranes by using centrifugation method because it is convenient and valuable for high-yield membrane protein separation and suitable for both comprehensive proteomics and targeting proteomics (61, 62). Crude membrane fraction in this study were chiefly plasma membranes but also contained endoplasmic reticulum, microsomes, and some mitochondria (63). It is also possible to enrich other membrane fractions by modification of the centrifugation protocol. If necessary, crude membrane fraction is subjected to gradient centrifugation for further purification (64). Affinity purifications, for example, surface biotinylation (21) or immunopurification (65), are applicable to enrich a specific population of the targeting membranes such as synaptic vesicles or exosomes, as well as organelles.

We strongly demonstrated that membrane washing is another critical step for improving membrane proteome data. Membrane washing is a simple and useful step. Notably, it is applicable for any of the aforementioned membrane isolation methods. While membrane washing has been established in membrane proteomics for a long time, it is not used very often recently. It is probably because of advances in analytical hardware or software, which give us more comprehensive proteome data, including more membrane proteins. Although alkaline-treatment has been used occasionally (66), on the other hand, urea treatment is rarely used for proteomics. No systematic comparison of the methods is available for all we notice. In this study, we compared side by side the efficiency of our methods, Urea Wash and Alkaline Wash, for membrane proteomics. Overall, both wash conditions were comparatively applicable for the enrichment of membrane proteins, especially TMD proteins. The success in high confidence protein identification is due to (at least) two rationales: (1) the soluble protein contaminants, which mask the signal of TMD protein, were removed by washing thereby, precursor signals of TMD proteins showed up and could be detected and (2) washing enriched the abundance of TMD proteins in the samples. They both yielded a high population of TMD proteins, although Urea Wash gave slightly better results for multiple TMD proteins and preserved protein complexes and clusters. Notably, the percentage of membrane proteins to the total identified proteins in No Wash, Urea Wash, and Alkaline Wash samples was 56.1%, 67.9%, and 74.6%, respectively. Alkaline Wash exhibited the highest percentage of membrane proteins to the total proteins because the number of total proteins in Alkaline Wash sample is smaller than others (Fig. 3*A*), suggesting that membrane washing with alkaline solution is harder and most likely removes more peripheral proteins that interact with membranes or membrane proteins, including functional interactions. The PPI network analysis also supports this hypothesis as we found that more PPIs were kept by Urea Wash than Alkaline Wash (Fig. 7). It was reported that high pH from sodium carbonate of alkaline-treatment resulted in depletion of some integral membrane proteins because free fatty acids released by saponification might act as mild detergents (67). This might be why the total intensity of peptides from Alkaline Wash sample before normalization was less than other samples (supplemental Fig. S3*A*). We propose that Urea Wash is suitable not only for comprehensive membrane proteomics but also for some specific proteomics, such as immunoprecipitation–MS and proximity-labeling MS, that is, APEX and BioID-MS, targeting membrane proteins. As shown in Figure 7, Urea Wash preserved more interacting functional proteins but may be enough to clean out most of the contaminants.

It is the advantage that urea itself does not disturb membranes but helps to solubilize the hydrophobic membrane proteins in water (68). Washing the membranes with urea is often applied to functional assays of membrane vesicles or purifications of membrane proteins because polytrophic membrane proteins, such as protein-conducting channels or transporters, are active even after urea treatment (19, 69, 70). Besides the advantage of Urea Wash in such biochemical assays, we demystified the merits of Urea Wash in comprehensive membrane proteomics. Urea Wash increased the identification numbers of particularly multiple TMD (TMD ≥10) proteins, such as SLC and ABC transporters. In every single cell of all organisms, such transporters play important roles for life. However, because of their minority in proteomes and their functional complexity, the linkage between proteomics and the transport functions is poorly reviewed. Over a quarter of 400 SLC genes are correlated with human diseases, although the studies of SLCs are relatively less compared with other gene families, which have similar stature (2). Transcriptome studies suggest that roughly 200 SLC transporters are expressed in the HEK293 cell line (71). We identified 87 SLCs in Urea Wash sample (supplemental Table S1). Because the numbers of mRNA transcriptions are not equal to that of protein expressions, our simple method may have identified about a half of SLC transporters in HEK293T cells. Urea Wash employs the merits of SLC studies by demonstrating a simple method to identify their expression profiles. In this study, we demonstrated the advantage of Urea Wash by the identification of SLC7A5 and SLC43A1 as the main leucine transporters and SLC6A9 as the major glycine transporter in HEK293T cells. Recently, we also utilized Urea Wash for the renal brush border membrane proteomics of the ischemia-reperfusion injury mouse model and identified SLC transporters responsible for an emerging clinical biomarker of acute kidney injury and chronic kidney disease (72).

Urea may also corporate the solubility and sustenance of the hydrophobic proteins in a hydrophilic environment (68). Urea-containing buffer is also compatible with trypsin digestion in the next step of bottom–up proteomics. In addition, urea does not significantly interfere with LC–MS/MS measurement. If necessary, urea can be removed prior to MS by common desalting techniques. Cautions for Urea Wash at high concentrations include (1) urea tends to aggregate during sample preparation at temperatures below 4 °C and (2) urea may cause a protein modification, called carbamylation, at elevated temperatures. Prolonged incubation of a protein sample with urea resulted in carbamylation, blocking protease digestion and affecting protein identifications and quantifications in MS analysis (8, 73, 74). Our study utilizes Urea Wash in the crude membrane as a model study for membrane proteomics. Nonetheless, Urea Wash is not limited to enrichments of plasma membranes. As mentioned previously, Urea Wash is applicable for enrichments of broad types of membranes, such as microsomes and organellar proteins. Notably, because Urea Wash is compatible with membrane protein functions, membrane samples after Urea Wash are applicable for combinational strategies of proteomics and biochemical approaches. For example, the same urea washed brush border membrane vesicles can be subjected to both functional enzymatic assays and quantitative proteomics.

Although recent advances in instruments and methods for sample preparation and data acquisition are drastic, the membrane enrichment protocol summarized here is simple, low cost, and requires no special equipment to unveil multiple TMD proteins and their PPI networks. Researchers who are not experts in proteomics can easily apply the protocol to their research. Moreover, multiple TMD proteins, such as transporters, still contain large numbers of uncharacterized or ignored molecules (2). The method is suitable for small-scale samples that require some additional biochemical assays to shed light on these orphan membrane proteins.

## Data Availability

All proteomics data have been deposited in Japan Proteome Standard Repository/Database (75): PXD027472, PXD027473, and PXD027474. The list of proteins and peptides were provided in supplemental Table S5 (Proteome of whole cell lysate and crude membrane of HEK293T cells), supplemental Table S6 (membrane proteome of HEK293T cells), and supplemental Table S7 (membrane proteome of renal brush border membrane vesicles from the mouse kidneys).

## Supplemental data

This article contains supplemental data (26).

## Conflict of interest

The authors declare that there is no conflict of interest.
