# Supplementary material for: Simple But Efficacious Enrichment of Integral Membrane Proteins and Their Interactions for In-Depth Membrane Proteomics
Source: Mol Cell Proteomics. 2022 Jan 25;21(5):100206. doi: 10.1016/j.mcpro.2022.100206 (PMC9062332; doi:10.1016/j.mcpro.2022.100206)
Supplement: Supplemental Figures S1–S4 [file mmc1.pdf]

Simple but efficacious enrichment of integral membrane proteins and their interactions for in-depth membrane proteomics

Pornparn Kongpracha[1,2], Pattama Wiriyasermkul[1,2], Noriyoshi Isozumi[3], Satomi Moriyama[2], Yoshikatsu Kanai[3], Shushi Nagamori[1,2,*]

[1]Department of Laboratory Medicine, The Jikei University School of Medicine, Tokyo, Japan

[2]Department of Collaborative Research for Biomolecular Dynamics, Nara Medical University, Nara, Japan

[3]Department of Bio-system Pharmacology, Graduate School of Medicine, Osaka University, Osaka, Japan

* Corresponding author. Email: snagamori@nagamori-lab.jp

Running title A sample preparation method for membrane proteomics

Supplemental Experimental Procedures

Isolation of Brush Border Membrane Vesicles (BBMVs) from Mouse Kidneys

The eight weeks old male mice of inbred strain C57BL/6J were anesthetized and exsanguinated. The kidneys were collected after perfusion with isotonic sodium chloride solution, quickly frozen in liquid nitrogen, and stored at -80 °C until the next process.

Preparation of Brush Border Membrane Vesicles (BBMVs) was performed as described with some modifications (26). The frozen kidneys were crushed twice for 10 sec at 1,700 rpm by Multi-beads shocker (Yasui Kikai Co., Osaka, Japan), and then homogenized in the homogenization buffer (0.05 g of sample/mL buffer; 20 mM Tris-HCl pH 7.6, 250 mM sucrose, 1 mM EDTA and protease inhibitor cocktail) using Potter-Elvehjem homogenizer. The homogenate was centrifuged at 1,000 ×g for 5 min at 4 °C, and the supernatant was centrifuged at 3,000 ×g for 5 min at 4 °C. The supernatant added with 11 mM $CaCl_2$ (from stock 1 M $CaCl_2$) was shaken on ice for 20 min followed by centrifugation at 3,000 ×*g* for 15 min at 4°C. After centrifugation, the supernatant was ultra-centrifuged at 438,000 ×*g* for 15 min at 4°C. The pellet was suspended in the homogenization buffer, and the $CaCl_2$ precipitation was repeated. The supernatant was ultra-centrifuged at 438,000 ×*g* for 15 min at 4 °C. The pellet was then resuspended in the buffer (20 mM Tris-HCl pH 7.6 and 250 mM sucrose), and an aliquot was subjected to a BCA protein assay. The procedure of washing BBMVs was performed as described in the *Experimental Procedures*; *Washing of Crude Membrane.*

LC-MS/MS and Data Analysis

For analysis of mouse BBMVs, the experiment was performed from one mouse kidney and one technical procedure for sample preparation. In LC-MS/MS analysis, the peptides were measured twice. The raw data was analyzed as described in *Experimental Procedures; Data Analysis* with some modifications. Raw data were analyzed using Proteome Discoverer 2.5 (Thermo Fisher Scientific, Waltham, MA) with Mascot 2.7 (Matrix Science, London, UK) against UniProt mouse database (released in January 2021) containing 83,714 protein sequence entries, including internal standards and trypsin sequences.

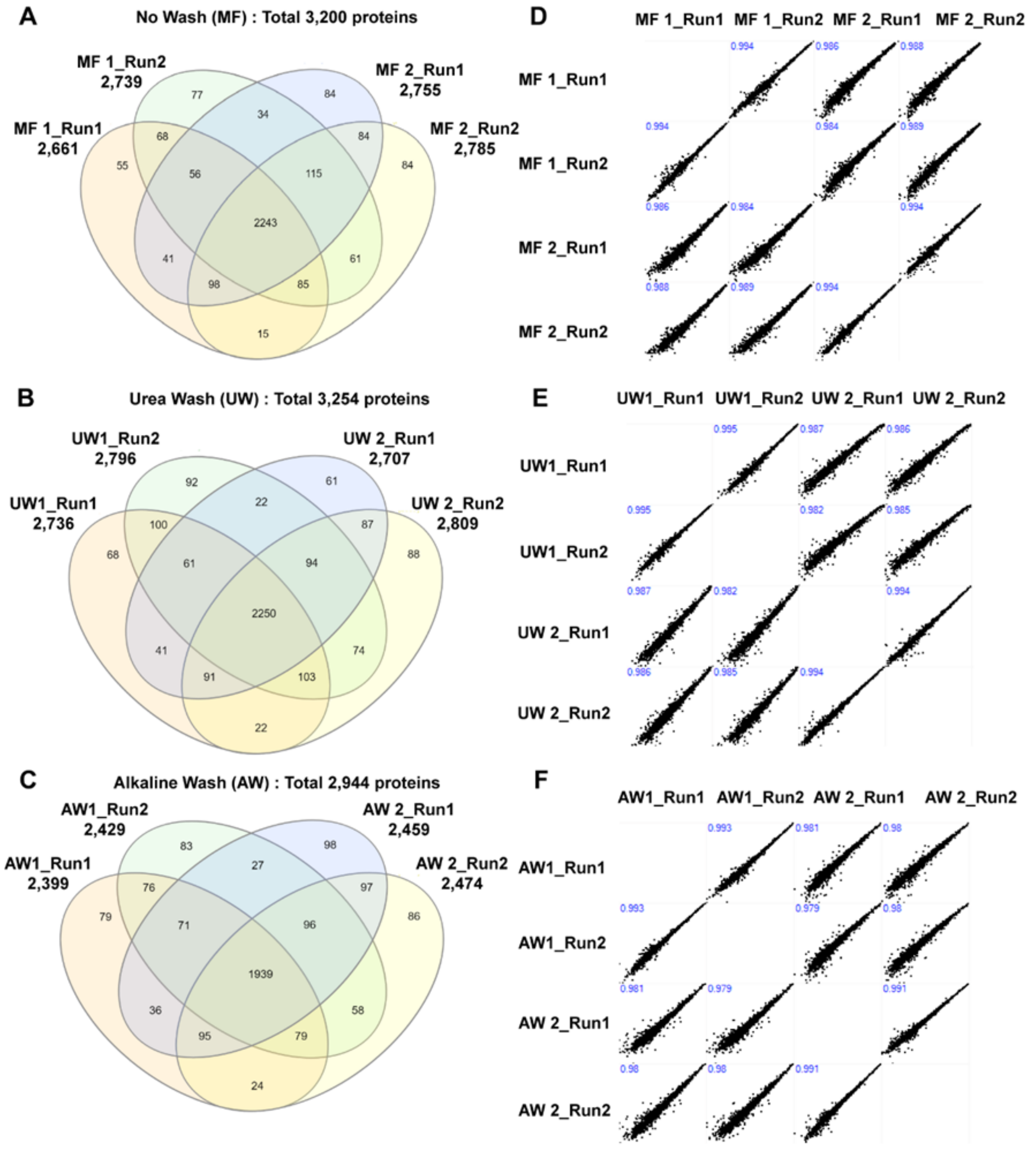

Kongpracha et al. supplemental Fig. S1

Supplemental Fig. S1. The total number of identified proteins and reproducibility of the experiments.

Crude membrane samples were prepared from two independent cell culture batches and subjected to No Wash (MF), Urea Wash (UW), and Alkaline Wash (AW). The peptides from each condition were measured twice by LC-MS/MS. *A, B* and *C,* Venn diagrams that show the overlap of identified proteins in samples without washing (No Wash) and with Urea Wash or Alkaline Wash. n = 4. *D, E,* and *F,* reproducibility of identified protein between four replicates determined by Pearson's correlation index.

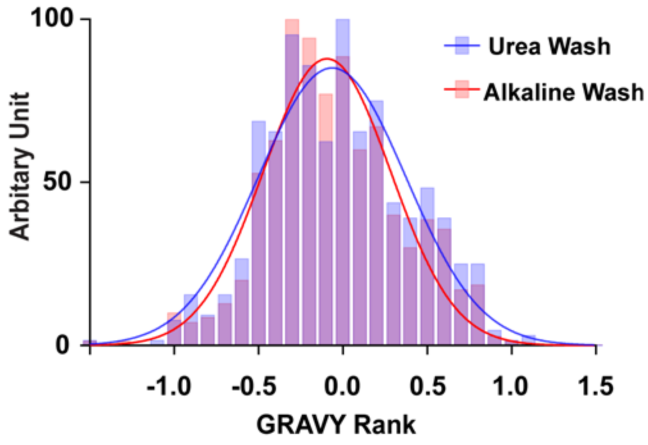

Kongpracha et al. supplemental Fig. S2

Supplemental Fig. S2. Evaluation of the hydrophobicity of proteome data from Urea Wash sample and Alkaline Wash sample.

Distribution curves show the numbers of the membrane proteins with GRAVY scores ≤ 0 and ≥ 0 from Urea Wash (blue) and Alkaline Wash (red) samples.

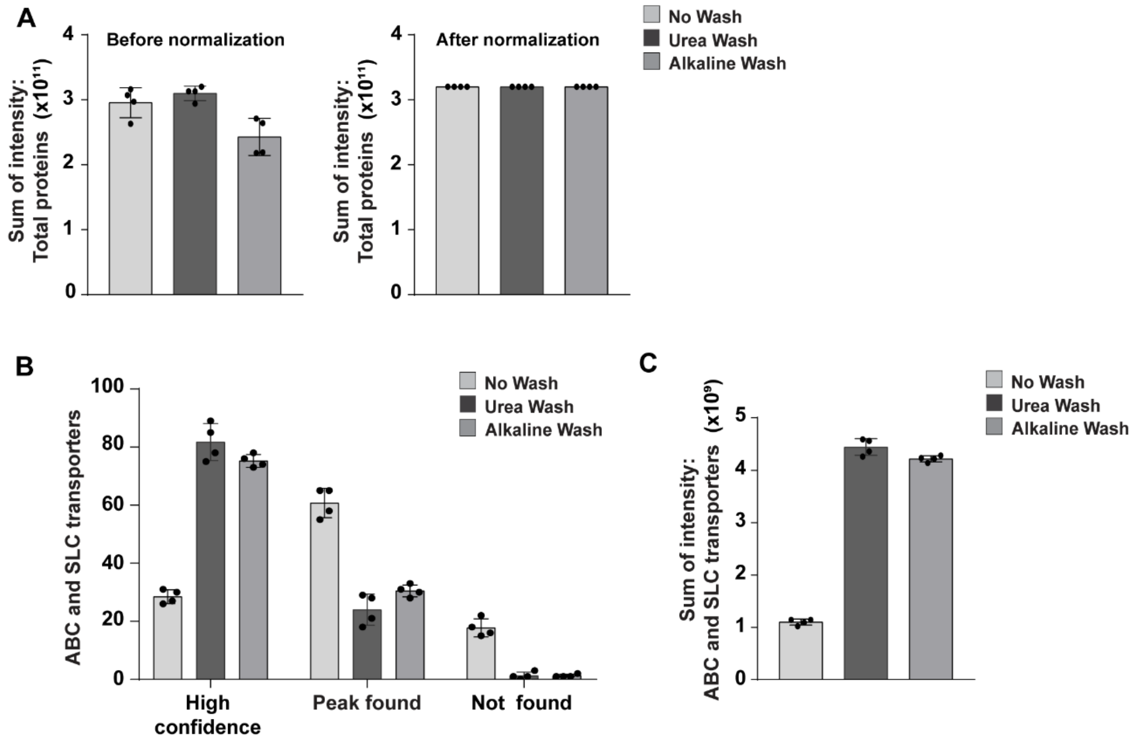

Kongpracha et al. supplemental Fig. S3

Supplemental Fig. S3. The impact of washing on the quantification of ABC and SLC transporters.

A, before and after normalization of the sum of intensity from all identified proteins. The bar graphs represent the average of the sum of intensity in samples without washing (No Wash) and with Urea Wash and Alkaline Wash. The sum of intensity was calculated from “High confidence” and “Peak found” proteins as defined in *Experimental Procedures*. The data indicate the mean ± SD, n = 4. *B*, the quantification of ABC and SLC transporters was evaluated based on quantification of precursor ion and availability of peptide spectra matched (PSMs) for the protein. The bar graphs represent the average numbers of ABC and SLC

transporters. Three types of proteins were shown: “High confidence”, “Peak found” (unidentified spectra), and “Not found” in samples without washing (No Wash) and with Urea Wash and Alkaline Wash. The data indicate the mean ± SD, n = 4. *C*, the quantity of ABC and SLC transporters. After normalization by total peptide amount, the relative abundance of ABC and SLC transporters was estimated from the sum of peptide intensities. The bar graphs represent the average of the sums of intensity in samples without washing (No Wash) and with Urea Wash and Alkaline Wash. The sum of intensity was calculated from “High confidence” and “Peak found” proteins. The data indicate the mean ± SD, n = 4.

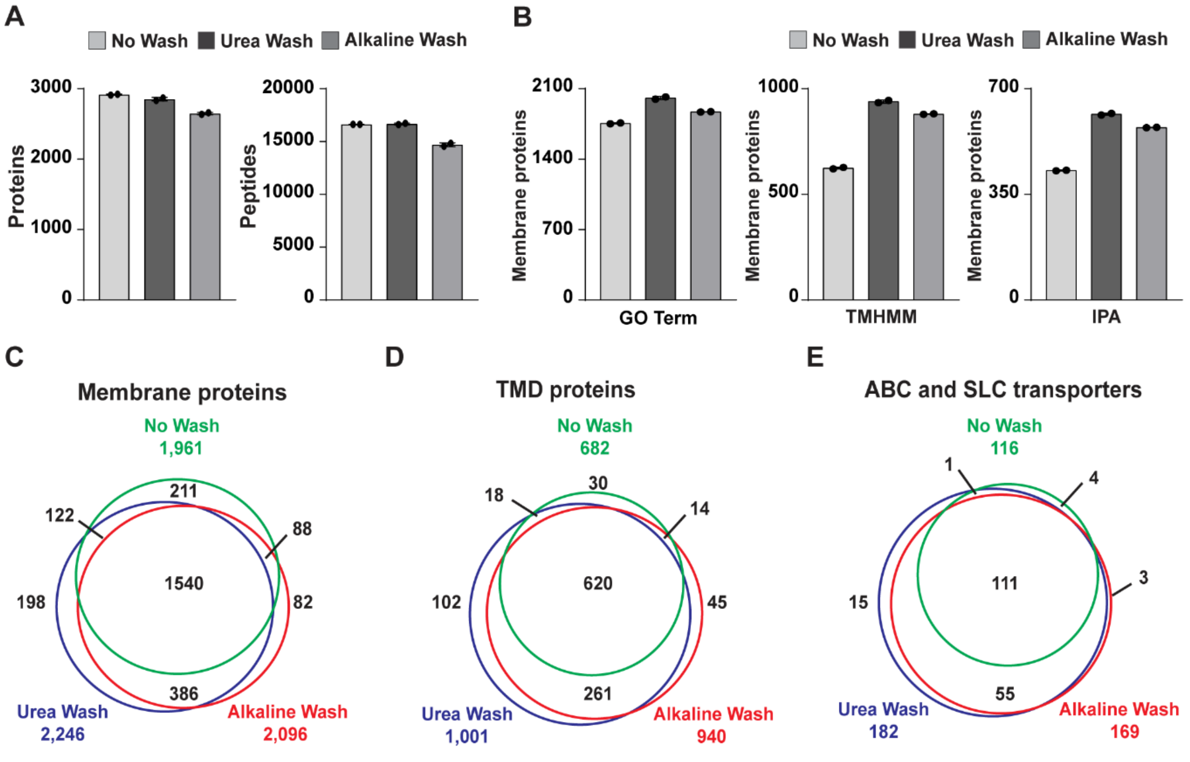

Supplemental Fig. S4. The impact of washing on membrane proteome of renal BBMVs from mouse kidneys.

A, bar graphs indicate the identified numbers of proteins and peptides from BBMVs in samples without washing (No Wash) and with Urea Wash and Alkaline Wash. The data indicate the mean ± SD, n = 2. *B*, bar graphs indicate numbers of membrane proteins annotated by GO, TMHMM, and IPA. The data represent mean ± SD, n = 2. *C*, Venn diagram indicates the average numbers of Membrane protein overlapping in samples without washing (No Wash) and with Urea Wash and Alkaline Wash. *D*, comparison of the numbers of TMD

proteins identified from different washing conditions. *E*, Venn diagram indicates the numbers of ABC and SLC transporters identified from different washing conditions.

List of Supplemental Tables

Supplemental Table S1: The identified ABC and SLC transporters in membrane proteomes of HEK293T cells.

Supplemental Table S2: The examples of protein complexes/clusters in membrane proteomes of HEK293T cells.

Supplemental Table S3: Protein-protein-interactions networks in Urea Wash sample.

Supplemental Table S4: Protein-protein-interactions networks in Alkaline Wash sample.

Supplemental Table S5: The identified proteins and peptides in whole cell lysate and crude membrane of HEK293T cells.

Supplemental Table S6: The identified proteins and peptides in membrane proteomes of HEK293T cells.

Supplemental Table S7: The identified proteins and peptides in membrane proteome of renal BBMVs from the mouse kidneys.
